# Case Report: Atypical HUS Presenting With Acute Rhabdomyolysis Highlights the Need for Individualized Eculizumab Dosing

**DOI:** 10.3389/fped.2022.841051

**Published:** 2022-02-23

**Authors:** Stefanie W. Benoit, Tsuyoshi Fukuda, Katherine VandenHeuvel, David Witte, Christine Fuller, Jennifer Willis, Bradley P. Dixon, Keri A. Drake

**Affiliations:** ^1^Division of Nephrology and Hypertension, Cincinnati Children's Hospital Medical Center, Cincinnati, OH, United States; ^2^Division of Bone Marrow Transplantation and Immune Deficiency, Cincinnati Children's Hospital Medical Center, Cincinnati, OH, United States; ^3^University of Cincinnati College of Medicine, Cincinnati, OH, United States; ^4^Division of Clinical Pharmacology, Cincinnati Children's Hospital Medical Center, Cincinnati, OH, United States; ^5^Division of Pathology, Cincinnati Children's Hospital Medical Center, Cincinnati, OH, United States; ^6^AdventHealth for Children, Orlando, FL, United States; ^7^Renal Section, Department of Pediatrics, University of Colorado School of Medicine, Aurora, CO, United States; ^8^Division of Pediatric Nephrology, University of Texas Southwestern Medical Center, Dallas, TX, United States

**Keywords:** atypical HUS, rhabdomyolysis, eculizumab, Bayesian modeling, therapeutic drug monitoring

## Abstract

**Background:**

Atypical hemolytic uremic syndrome (aHUS) is an ultra-rare orphan disease caused by dysregulated complement activation resulting in thrombotic microangiopathy. Although complement-mediated endothelial injury predominantly affects the renal microvasculature, extra-renal manifestations are present in a significant proportion of patients. While eculizumab has significantly improved the morbidity and mortality of this rare disease, optimizing therapeutic regimens of this highly expensive drug remains an active area of research in the treatment of aHUS.

**Case Presentation:**

This report describes the case of a previously healthy 4 year-old male who presented with rhabdomyolysis preceding the development of aHUS with anuric kidney injury requiring dialysis. Clinical stabilization required increased and more frequent eculizumab doses compared with the standardized weight-based guidelines. In the maintenance phase of his disease, pharmacokinetic analysis indicated adequate eculizumab levels could be maintained with an individualized dosing regimen every 3 weeks, as opposed to standard 2 week dosing, confirmed in this patient over a 4 year follow up period. Cost analyses show that weight-based maintenance dosing costs $312,000 per year, while extending the dosing interval to every 3 weeks would cost $208,000, a savings of $104,000 per year, relative to the cost of $72,000 from more frequent eculizumab dosing during his initial hospitalization to suppress his acute disease.

**Conclusion:**

This case exemplifies the potential of severe, multisystem involvement of aHUS presenting with extra-renal manifestations, including rhabdomyolysis as in this case, and highlights the possibility for improved clinical outcomes and higher value care with individualized eculizumab dosing in patients over the course of their disease.

## Introduction

Atypical hemolytic uremic syndrome (aHUS) is a progressive, life-threatening thrombotic microangiopathy (TMA) that is caused by dysregulation of the complement system, a component of the innate immune system made up of distinct plasma and membrane-bound proteins that, when activated, react with one another in a pro-inflammatory cascade. Eculizumab (Soliris^®^, Alexion, Boston, MA) is a humanized monoclonal antibody that binds with high affinity to the complement protein C5, interrupting the terminal pathway of the complement cascade and preventing formation of the membrane attack complex, C5b-9. Prior to eculizumab, therapy relied on supportive measures including plasma exchange and plasma infusion, although trials of these interventions in patients with aHUS were few and outdated (1). Depending on the underlying complement abnormality, as many as 70% of patients lost renal function or died during the presenting episode, and the majority of patients developed end-stage renal disease within 2 years of presentation (2). Eculizumab was FDA approved for treatment of aHUS in 2011 and has changed the natural history of the disease to one of maintained renal function with chronic complement suppression (3, 4).

Eculizumab remains one of the most expensive medications currently available, with an acquisition cost of ~$6,000 per 300 mg vial (per pharmaceutical prices from the Office of Procurement, Acquisition and Logistics, OPAL, at the United States Department of Veteran's Affairs). This has led to increased oversight and heightened review of its use as a high-cost medication (5). Standard maintenance dosing to suppress disease activity is weight based, ranging from infusion of 300 mg every 2 weeks for patients >10 kg to 1,200 mg every 2 weeks for patients > 40 kg. Thus, the annual cost of maintenance therapy in a patient ≥40 kg is $576,000 for drug alone, not including intravenous access and infusion costs, patient time, and quality of life. Recently, there have been a number of reports in the literature describing various factors that affect eculizumab pharmacokinetics, including varying disease severity as well as other medical co-morbidities, further highlighting the need to individualize treatment approaches (6–9). However, there remains only limited, and somewhat variable, reports in the literature to guide such approaches for patients with aHUS, For example, one published strategy for cost reduction that simply reported holding eculizumab and monitoring for relapse. This study found that thirty percent of patients relapsed within 6 weeks of cessation, demonstrating the risk of end organ damage and severe illness that this method poses to aHUS patients (10). However, pharmacokinetic and pharmacodynamic studies in patient with TMA after hematopoietic stem cell transplantation have been instrumental in developing algorithms to specifically guide eculizumab therapy (6, 11), which have subsequently been examined in patients with aHUS and paroxysmal nocturnal hemoglobinuria. For instance, Gatault et al. demonstrated that while intra-individual variability of eculizumab trough concentrations was low with a coefficient of variation <25%, the inter-individual variability was 63%, with troughs ranging from 55 ± 12 to 733 ± 164 μg/mL (12). Given the recommended eculizumab trough level to maintain disease control has been reported as 50–100 μg/mL (13), this study showed that there were some patients with troughs at borderline therapeutic levels while others had over 7 times the required amount. Additionally, Ardissino et al. published a strategy of weaning eculizumab based on maintaining global classical complement activity to <30% (14). While no renal or extra-renal relapse was detected during the study period, they found that 25% of the complement activity measurements were higher than their target, suggesting that a significant portion of their patients may have been at risk of relapse. Overall, these studies highlight the continued need and critical importance of optimizing therapeutic approaches in patients treated with eculizumab.

This report describes an unusual presentation of aHUS with skeletal muscle involvement (confirmed by biopsy-proven TMA) and additionally highlights how individualizing eculizumab dosing via pharmacokinetic modeling can be utilized to improve clinical outcomes and promote cost savings over time in patients diagnosed with aHUS.

## Case Description

A 4 year-old, previously healthy Caucasian male, was evaluated in the emergency department for an acute onset of bilateral leg pain. Work up included a normal complete blood count, femur and tibia/fibula x-rays, and hip ultrasound. His creatine kinase (CK) was found to be elevated (854 Units/L), and he was discharged home with supportive care for presumed viral myositis. Over the next week, he developed a new-onset rash on his face and neck, persistent fatigue, and low-grade fevers (100.6 F). He was subsequently admitted to the hospital with persistent muscle pain, increased weakness, and an increasing CK (11,000 Units/L). On admission, labs showed a hemoglobin (Hgb) of 12.6 g/dL, platelets 98,000, BUN 21 mg/dL, creatinine 0.3 mg/dL, albumin 3.1 g/dL, and urinalysis showed >300 mg/dL protein with moderate blood. Rheumatology was consulted, and he empirically received intravenous immunoglobulin.

**Figure 1:**
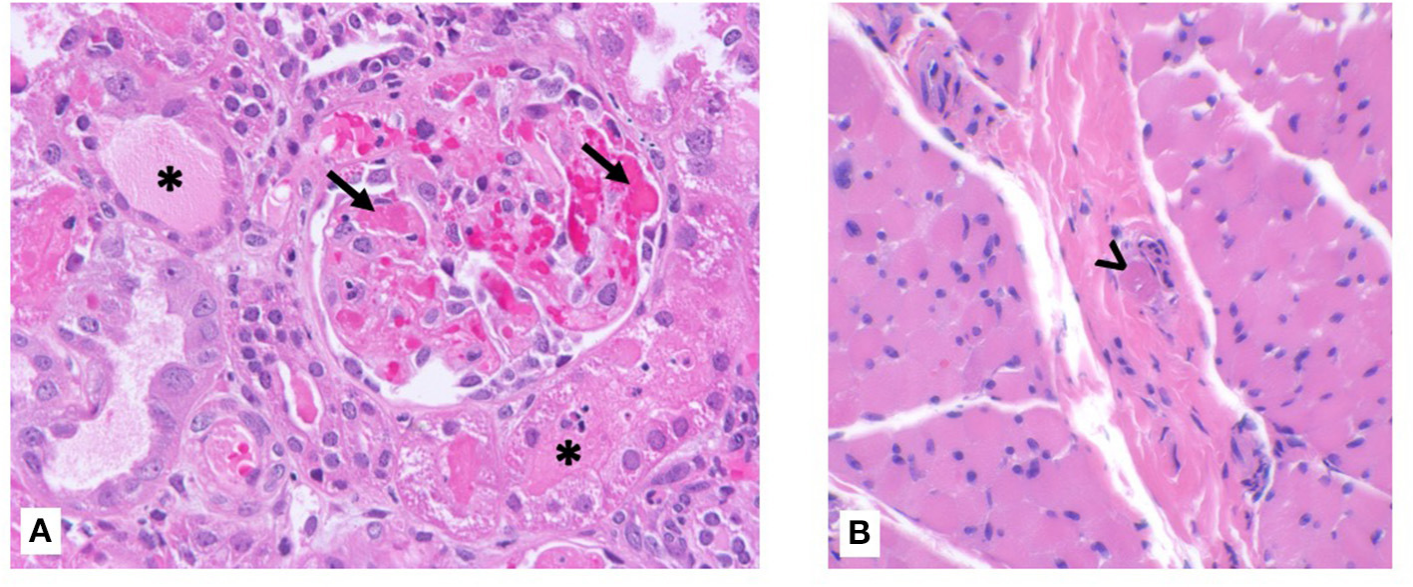
Kidney and muscle biopsy specimens. **(A)** Renal biopsy showing fibrin microthrombi within glomerular capillaries (arrow) with possible early crescent formation. Tubules show diffuse injury with cytoplasmic vacuolization and focal casts (asterisk). Hematoxylin & eosin, ×400 magnification. **(B)** Skeletal muscle biopsy showing intravascular microthrombi within a perimysial vessel (arrowhead). Hematoxylin & eosin, ×400 magnification.

On day 2 of hospitalization, he developed anemia (Hgb 9.9 g/dL) with schistocytes, worsened thrombocytopenia (platelets 28,000), increased creatinine (0.6 mg/dL), and elevated blood pressures, consistent with hemolytic uremic syndrome. There was no history of diarrhea, thus Shiga toxin testing was not performed. ADAMTS13 activity was normal. He was given a weight-based induction dose of eculizumab 600 mg on hospital day 4. Given this, he also received initial pneumococcal and meningococcal vaccines and was started on penicillin prophylaxis. Kidney function continued to decline, and on hospital day 5, renal and muscle biopsies were performed at the same time as placement of hemodialysis and peritoneal dialysis catheters. He was initiated on hemodialysis post-operatively with a BUN of 142 mg/dL and creatinine 3.1 mg/dL. The renal biopsy revealed TMA with severe acute tubular necrosis (Figure 1A). The muscle biopsy also demonstrated TMA in a skeletal muscle vessel (Figure 1B).

**Figure 2:**
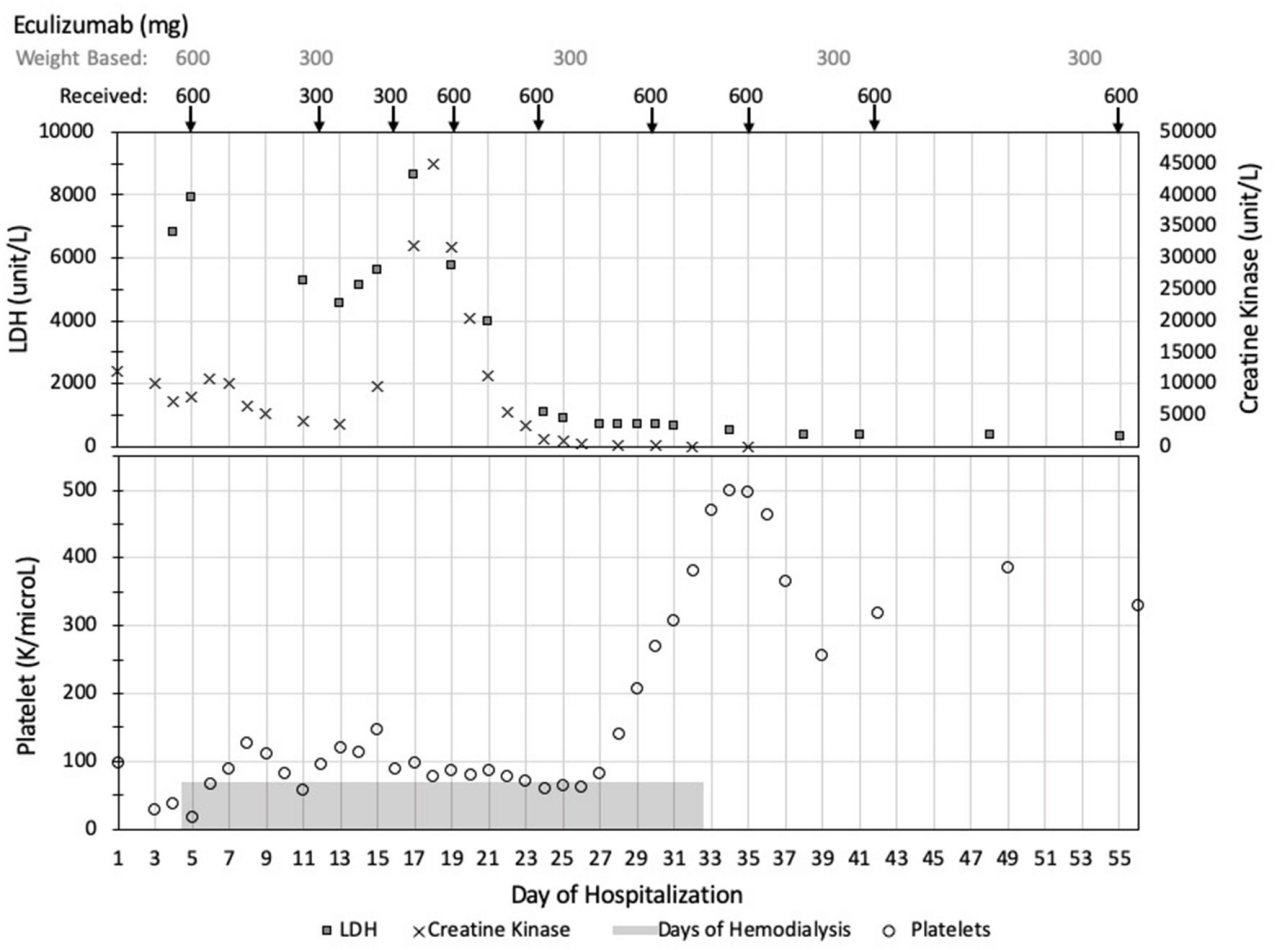
Summary of clinical values and eculizumab dosing. Data is presented in chronological order with days of hospitalization listed across the bottom of the figure. Eculizumab dosing started on day of hospitalization 4. The weight-based recommended dosing of eculizumab is listed across the top of the figure with the actual doses received reported directly below. TMA activity is demonstrated with elevated LDH (squares) and low platelets (circles). In his unique case, his rhabdomyolysis, as demonstrated by elevated CK levels (crosses), paralleled his TMA activity. TMA, thrombotic microangiopathy; LDH, lactate dehydrogenase; CK, creatine kinase.

After his initial induction dose of eculizumab, the patient's platelets peaked at 126,000. However, 6 days after his first dose, his platelets trended down to 58,000. He received a second dose of eculizumab 300 mg per standard weight-based dosing given that his weight was 18 kg. Platelets increased to 146,000 but dropped to 88,000 just 3 days after his second eculizumab dose. He received a supplemental dose of 300 mg and then was maintained on weekly infusions of 600 mg. Despite additional supplemental and larger doses of eculizumab, as detailed in Figure 2, his course waxed and waned with two worsening episodes of rhabdomyolysis.

On hospital day 24, he transferred institutions for further management of his myopathy and aHUS that appeared to be refractory to standard eculizumab dosing. The patient underwent an extensive work up, with genetics studies negative for the 10 most commonly associated aHUS genes (*C3, CFB, CFH, CFHR1* full sequence and deletions/duplications, *CFHR3* full sequence and deletions/duplications, *CFHR5, CFI, DGKE, MCP, THBD*). Homocysteine and methylmalonic acid levels were normal, ruling out cobalamin C-associated aHUS. Additional work up for rheumatological, infectious, metabolic, and neuromuscular disease were all negative. Specifically, this included a normal complement profile (except for a mildly elevated C5, C6, and C1 inhibitor consistent with an acute phase reaction) and included a normal properdin, Bb, Factor B, Factor H, Factor I, and negative Factor H autoantibody. His muscle biopsy was reviewed by multiple pathologists, with findings consistent with ischemic muscle injury and TMA and no signs of primary muscle pathology or metabolic disorder, juvenile dermatomyositis, or another autoimmune myopathy. Additional rheumatologic/immunologic work up also included: ANA titer 1:80 with repeat ANA negative, ANCA negative, and negative lupus panel. X-linked adrenoleukodystrophy testing (very long chain fatty acids, phytanic acid, pristanic acid) was normal.

**Figure 3:**
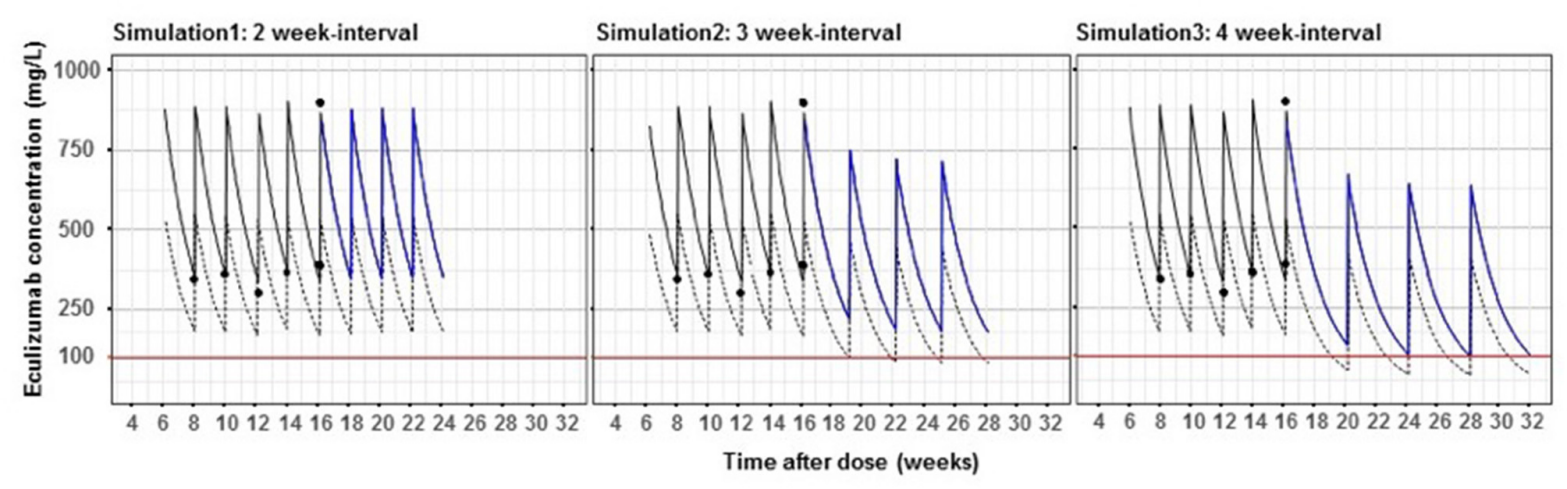
Bayesian pharmacokinetic modeling of maintenance eculizumab dosing in an aHUS patient. Dashed lines show the population pharmacokinetic curve in aHUS patients based on mean pharmacokinetic parameters in Federal Drug Administration approval documents. Solid lines show this patient's pharmacokinetic profile based on five eculizumab levels (black circles). Red lines show the recommended concentration of eculizumab to control aHUS. Blue lines are simulated pharmacokinetic profiles of this patient with 2, 3, and 4 week-interval dosing.

The patient required intermittent hemodialysis over a 3 week period. Subsequently, his platelets normalized, anemia remained stable, and creatinine trended down to 0.4 mg/dL. Guided by his laboratory values, he was weaned to the standard weight-based maintenance dosing frequency of eculizumab 600 mg every 2 weeks with eculizumab trough levels maintained >100 mcg/mL, soluble membrane attack complex (sC5b-9) below the normal limit of 250 ng/mL, and suppressed CH50 below limits of detection. He completed his pneumococcal and meningococcal vaccination series (including PPSV23, MenACY, and MenB) due to the need for long-term eculizumab treatment. Given our institution's experience with eculizumab dosing in hematopoietic stem cell transplant associated TMA using Bayesian pharmacokinetic modeling (6), we applied the same personalized dosing strategy to this patient. Bayesian estimators, based on pharmacokinetic parameters with the incorporation of a patient's individual data, have been developed to estimate therapeutic dosing. As shown in Figure 3, the patient's pharmacokinetic modeling suggested he could be safely maintained on every 3 week dosing schedule. After extensive discussions with the family, he was transitioned to this regimen with labs initially drawn prior to each infusion then spaced to every 3–4 months. Follow up over the last 3 years of monitoring has shown levels of sC5b-9 below the upper limits of normal (250 ng/mL), suppressed CH50 below the limit of detection, and eculizumab levels ranging from 120 to 280 mcg/mL, with no clinical relapses of his aHUS. Standard maintenance dosing on a 2 week regimen for this patient is estimated to cost $312,000 per year, while increasing the dosing interval to every 3 weeks costs $208,000, a savings of $104,000 per year.

## Discussion

Diagnostic criteria for aHUS include microangiopathic hemolytic anemia with increased lactate dehydrogenase, decreased haptoglobin, thrombocytopenia, and organ dysfunction in the setting of normal ADAMTS13 activity and the absence of Shigatoxin-associated *E*. coli infection. Extrarenal manifestations, including cerebral, cardiac, ocular, neurologic, gastrointestinal, and pulmonary involvement, occur in 20% of patients (15). Acute rhabdomyolysis with muscle TMA as the presenting symptoms of aHUS is extraordinarily rare, although it has been previously reported (16). It is unclear if the inciting event in our case was a viral myositis, which unveiled his susceptibility to aHUS and resulted in TMA of the muscle and kidneys, or if his aHUS truly manifested first in the skeletal muscle prior to involvement of other organ systems as suggested by his disease course and muscle biopsy findings.

The model we applied to individualize eculizumab dosing uses Bayesian fitting with population parameters targeting eculizumab levels >100 mcg/mL (6). It was originally developed using a substantial population of patients with hematopoietic stem cell transplant (HSCT)-associated TMA, a similar form of TMA characterized by severe, time-limited activation of the complement cascade. In this closely related disease entity, the authors found that the pre-treatment sC5b-9 level, a marker of terminal complement activation, was a key determinant of the eculizumab concentration-time profile, with children with higher sC5b-9 levels requiring more frequent dosing to achieve initial blockade (6). Moreover, once complement blockade was achieved and inflammation controlled, which can be ensured by measurement of CH50 values, less frequent eculizumab dosing was sufficient to maintain effective blockade (6). When the predictive performance of this model was evaluated, 94.7% of 38 observed concentrations taken up to 4 days after the initial treatment, were within ±20% of the model-predicted concentrations. Notably, the studies described above targeting eculizumab levels > 100 mcg/mL and use of CH50 and sC5b-9 as pharmacodynamic markers of adequate complement blockade have only recently been published in the last 3–5 years, prior to many of the consensus statements and published guidelines for the treatment of aHUS (2, 17) and the use of these monitoring parameters continues to vary by institution.

Our patient initially required an increase in dose amount and frequency to achieve a sufficient and sustained clinical response. This may have been due to innate, profound complement activation and dysregulation, as has been documented in the HSCT-associated TMA population as described above. This may also be due in part to the dose of intravenous immunoglobulin he received prior to eculizumab therapy, as it has been shown that patients receiving intravenous immunoglobulin have increased clearance of monoclonal antibodies via the neonatal Fc receptor, which results in significantly lower mean serum levels of eculizumab (18). Other factors, such as gastrointestinal bleeding and severe proteinuria, have also been suggest to affect eculizumab clearance (8, 11, 19). Unfortunately, one limitation of our report is that eculizumab levels, sC5b-9, and CH50 were not monitored initially in the patient's hospitalization in correlation with his worsening thrombocytopenia, though his clinical course supports that he subsequently responded to increased doses of eculizumab.

Whatever the underlying reason for the increased requirement of eculizumab for disease suppression, our approach may appropriately raise concerns about the significant increase in cost of initial therapy. The cost of the eculizumab doses required to suppress our patient's early, severe disease was $72,000, which may have been further personalized through monitoring of sC5b-9, CH50, and eculizumab levels during his acute presentation as described above. Nevertheless, we offer an alternative conclusion that prevention of lifelong severe morbidity, including prolonged ICU stay and hospitalization, chronic kidney disease, dialysis, and renal transplant, far outweighs the cost of adequate early disease suppression. In addition, individualized weaning of eculizumab in the maintenance phase may be safely executed and provide significant cost savings. Similar considerations also apply to the newest terminal complement inhibitor—ravulizumab, which binds with greater affinity to the neonatal Fc immunoglobulin receptor and with altered affinity to the C5 molecule within the acidified endosome, extending the dosing interval to every 8 weeks. However, the same complement tests (i.e., CH50 and sC5b-9) used for eculizumab may not consistently indicate adequate complement blockade (free C5 < 0.5 μg/mL) in monitoring therapy with ravulizumab (20). While this is an active area of research (21, 22), recent studies suggest that ravulizumab offers a cost-savings and improved quality of life with less frequent dosing compared to the current cost of eculizumab (23) and adapted monitoring parameters (such as free C5 assays) are expected to be useful in individualizing the dosing of this drug as well.

## Patient Perspective

During the whole process, the patient and his parents were informed about treatment options, risk and possibility of relapse. They were aware of the complexity of his unusual case, and the parents provided written informed consent along verbal assent from the patient for the publication of his case.

## Data Availability Statement

The original contributions presented in the study are included in the article/supplementary material, further inquiries can be directed to the corresponding author/s.

## Ethics Statement

Written informed consent was obtained from the minor(s)' legal guardian/next of kin for the publication of any potentially identifiable images or data included in this article.

## Author Contributions

SB, TF, BD, and KD: research idea and study design and data analysis/interpretation. BD and KD: supervision. SB and KD: manuscript drafting. SB, BD, and KD: manuscript reviewing. All authors contributed to the article and approved the submitted version.

## Conflict of Interest

BD has participated in consulting agreements with Apellis Pharmaceuticals and Alexion Pharmaceuticals. The remaining authors declare that the research was conducted in the absence of any commercial or financial relationships that could be construed as a potential conflict of interest.

## Publisher's Note

All claims expressed in this article are solely those of the authors and do not necessarily represent those of their affiliated organizations, or those of the publisher, the editors and the reviewers. Any product that may be evaluated in this article, or claim that may be made by its manufacturer, is not guaranteed or endorsed by the publisher.
